# The effect of an elemental diet on oral mucositis of esophageal cancer patients treated with DCF chemotherapy: a multi-center prospective feasibility study (EPOC study)

**DOI:** 10.1007/s10388-018-0620-1

**Published:** 2018-05-31

**Authors:** Yoshihiro Tanaka, Takao Ueno, Naoya Yoshida, Yasunori Akutsu, Hiroya Takeuchi, Hideo Baba, Hisahiro Matsubara, Yuko Kitagawa, Kazuhiro Yoshida

**Affiliations:** 1 Department of Surgical Oncology, Graduate School of Medicine, Gifu University, 1-1 Yanagido, Gifu, Gifu 501-1194 Japan; 2 Dental Division of National Cancer Center, Tokyo, Japan; 3 Department of Gastroenterological Surgery, Graduate School of Medical Sciences, Kumamoto University, Kumamoto, Japan; 4 Department of Frontier Surgery, Graduate School of Medicine, Chiba University, Chiba, Japan; 5 Department of Surgery, Graduate School of Medicine, Keio University, Tokyo, Japan

**Keywords:** Chemotherapy, Oral mucositis, Esophageal carcinoma, DCF, Central review system

## Abstract

**Purpose:**

Oral mucositis (OM) is one of the most uncomfortable adverse events experienced by cancer patients undergoing chemotherapy. Previous reports have revealed that the oral administration of an elemental diet (ED) may prevent OM. However, the incidence of OM has not been accurately determined by specialized diagnostic methods and the effects of an ED on OM remain unclear. We investigated the dose that could feasibly be administered and its effects with regard to the suppression of OM in esophageal cancer patients undergoing chemotherapy.

**Methods:**

We performed a prospective multi-center feasibility study of the administration of an ED (160 g/day) with 2 cycles of docetaxel/cisplatin/5-FU (DCF) chemotherapy. We assessed compliance to the ED for 49 days and the incidence of OM according to the amount of the ED that was orally administered. The incidence of OM was graded by a dental specialist who was experienced in dental oncology using a central OM review system.

**Results:**

Fourteen of 20 patients (70%) were able to complete the orally administered ED (160 g/day) during the course of chemotherapy. Three patients (15%) could not take the ED orally for 9, 14, and 21 days, respectively, while 1 patient (5%) took the ED orally at an average dose of 80 g/day for 35 days. The remaining 2 patients (10%) could not take the 80 g/day dose for 11 and 12 days, respectively. The incidence of grade ≥ 2 OM in the ED completion group (15.4%, 2 of 13 patients) was significantly lower than that in the non-completion group (66.7%, 4 of 6 patients) (*p *= 0.046).

**Conclusions:**

An ED might be a one of the test treatment to reduce the incidence of OM in esophageal cancer patients treated with DCF and should be evaluated in further randomized study.

**Clinical trial:**

The date of submission: Dec 08th, 2017.

**Electronic supplementary material:**

The online version of this article (10.1007/s10388-018-0620-1) contains supplementary material, which is available to authorized users.

## Introduction

Advances in medical device development have reduced the incidence of complications after surgery for esophageal cancer; however, even in patients in whom curative resection is achieved, the 5-year survival rate is only 20–36% [1]. In patients with operable esophageal cancer, there is evidence to support the use of preoperative chemotherapy or chemoradiation [2, 3]. Meanwhile, unresectable or metastatic esophageal cancer has also been treated with chemotherapy [4]. Chemotherapy can significantly improve the clinical outcomes of cancer patients, but it can also result in serious adverse effects [5, 6].

The current standard neoadjuvant chemotherapy for esophageal cancer is 5-fluorouracil (5-FU)/cisplatin (FP) [7]. Moreover, more effective chemotherapy regimens using docetaxel/cisplatin/5-FU (DCF) have been reported [8]. However, this regimen is associated with an increased incidence of severe adverse effects, including hematological and gastrointestinal (GI) toxicities. To overcome such adverse events, we recently showed that a modified DCF regimen can reduce the hematological toxicity of the regimen [9, 10]; however, oral mucositis (OM) was often observed. GI toxicities caused by chemotherapy can negatively affect a patient’s nutritional status and result in the discontinuation of chemotherapy. OM—one of the most common GI toxicities—results in increased pain, difficulty in swallowing, nutritional compromise, and an increased risk of infection.

Taxanes, platinum-containing drugs, and FUs are all reported to cause mucosal damage, with an incidence of up to 70% [11, 12]. However, several countermeasures to prevent OM with chemo(radio)therapy have been reported [13–15], those effects might not be sufficient for multidrug anticancer agents. A report indicated that the oral glutamine (Gln) administration reduced the duration and severity of OM after cytotoxic cancer chemotherapy. [16]. Thus, we previously conducted a randomized phase II trial to study the effects of Gln plus one pack (80 g) of elemental diet {ED [(Elental^®^; EA Pharma Co., Ltd.)]}/day: total Gln 8862 mg/day or Gln alone: 8910 mg/day compared to no prevention of OM in patients with esophageal cancer undergoing chemotherapy including FP and triplet regimen [17]. Only the Gln plus an ED group showed a significant preventive effect on the development and severity of OM. We concluded that the oral administration of Gln plus an ED [one pack (80 g)] may prevent OM. Even though the total amount of Gln administered to the two treatment groups: Gln plus an ED group and Gln group was nearly equal, only the addition of the ED group had a significant preventive effect against OM. Thus, the ED was thought to have an inhibitory effect against OM due to other amino acids such as histidine which has also anti-inflammatory effect [18] like Gln contained in the ED. In addition, the mechanism of the effects of the ED might involve the maintenance of the mucosal integrity, which is indicated by significant higher levels of plasma diamine oxidase (DAO) activity [17]. In the present study, we evaluated the preventive effect of ED alone against OM in patients undergoing DCF chemotherapy. Considering that Gln formulation was not added in the combination of 1 pack of an ED (80 g) this time and that OM was likely to occur with the DCF regimen, we thought that ED would require at least more than 2 packs (160 g). On that occasion, in consideration of the situation that it was not easy to drink 1 pack (80 g)/day of an ED during chemotherapy in Ogata et al.’s report, we thought that 2 packs (160 g) were appropriate this time. So, we set the dosage of the ED to 2 packs (160 g)/day, which was twice the dosage of our previous report. We assessed compliance to the ED and the incidence of OM according to the amount of the ED that was orally administered.

## Methods

### Study design

#### Endpoints and methods

The primary endpoint of this study was the completion rate of an orally administered ED 2 packs (160 g/day) during 2 cycles of DCF chemotherapy. The secondary endpoints were the incidence of OM (CTCAE ver. 3.0) in patients who completed taking the orally administered ED (160 g/day; completion group) and in those who could not complete it (non-completion group); the rate of weight fluctuation; DAO activity, which is a reliable indicator of intestinal mucosal integrity; the turnover rate of plasma proteins (prealbumin, lymphocyte count), which was used as an indicator of the nutritional status per compliance with the orally administered ED; adverse events other than OM (CTCAE ver. 3.0); and the objective response rate to chemotherapy.

In the present study, the patients were scheduled to receive an ED at a dose of 2 packs (160 g/day). The ED was administered orally 1 week before chemotherapy and was continued during chemotherapy for a total of 49 days. The rate of weight fluctuation, DAO activity, prealbumin level, and lymphocyte count were measured on days 1, 8, and 15 in each of the 2 chemotherapy cycles. All patients received preventative oral care before chemotherapy.

We constructed a central review system (CRS) to judge the oral environment. The CRS judge assessed the oral mucosa of each patient before chemotherapy and on day 1, 8, and 15 of each of the 2 cycles of DCF using the CRS. Oral and maxillofacial surgeons at each institution used instruments to examine the oral cavity. Six photographs (that included the posterior surface of the upper and lower lips, right and left buccal mucosa, and right and left lingual surfaces) were taken using a specialized intraoral imaging camera (Online Resource 1) and transmitted as a 4 MB electronic file to the data server prior to the diagnosis of OM. In each case, OM was graded by a CRS judge (a dental specialist who was experienced in dental oncology) who did not belong to the institutions with registered patients, and who was unaware of the patients’ background information.

OM was graded according to CTCAE ver 3.0 [19], based on the results of a clinical examination, as follows: Grade 1, erythema of the mucosa; Grade 2, patchy ulcerations or pseudomembranes; Grade 3, confluent ulcerations or pseudomembranes, bleeding with minor trauma; Grade 4, tissue necrosis, significant spontaneous bleeding, life-threatening consequences; and Grade 5, death.

Patients were enrolled from four institutions: Gifu University Hospital, Keio University Hospital, Kumamoto University Hospital, and Chiba University Hospital.

#### Eligibility criteria

Patients who were > 18 years of age at the time of registration, and who had histologically or cytologically confirmed Stage II/III esophageal squamous cell carcinoma or adenocarcinoma were included in the present study. The staging of all patients was defined by the guidelines of the Japanese Society for Esophageal Disease (10th edition). The other inclusion criteria were as follows: an Eastern Cooperative Oncology Group performance status of 0–1; a life expectancy of > 12 weeks; and adequate liver, bone marrow, renal, and cardiovascular functions [serum bilirubin ≤ 1.5 mg/dl; neutrophil count ≥ 1500/mm^3^; serum aspartate aminotransferase and alanine aminotransferase levels ≤ twice the upper limit of normal range; platelet count ≥ 10 × 10^4^/mm^3^; hemoglobin ≥ 8.0 g/dl; and creatinine ≤ 1.2 mg/dl (or creatinine clearance > 60 ml/min)].

Patients who had previously received chemotherapy for malignant disease were excluded from the study. The other major exclusion criteria were as follows: serious concomitant illness, symptomatic infectious disease, severe allergy, peripheral neuropathy, or uncontrolled diabetes mellitus.

#### The treatment regimen and operation, and the assessment of the tumor response and adverse events

DCF chemotherapy consisted of a 1-h intravenous (i.v.) infusion of docetaxel (70 mg⁄m^2^), a 2-h infusion of cisplatin (70 mg⁄m^2^) on Day 1, and a continuous i.v. infusion of 5-FU (750 mg⁄m^2^⁄day) on days 1–5. This regimen was repeated every 3 weeks. This regimen was administered as preoperative chemotherapy to all patients. Two cycles of this regimen were administered within 2 weeks after registration in this study. Prophylactic antibiotics were routinely used for 10 days from Day 6 of each cycle. Prophylactic administration of granulocyte-colony stimulating factor (G-CSF) on the chemotherapy day was not allowed, and G-CSF was permitted to administer when neutropenia or fever occurred. After an interval of 4–6 weeks from the completion of chemotherapy, radical esophageal resection and lymphadenectomy were scheduled by open thoracotomy or video-assisted surgery.

The tumor response was assessed according to the Response Evaluation Criteria in Solid Tumors guidelines [20] after the second cycle of chemotherapy and 4 weeks later. A barium meal study, endoscopy, ultrasonography, and computed tomography were used to evaluate the response of measurable lesions. A complete response (CR) was defined as the complete disappearance of all clinically detectable malignant disease; a partial response (PR) was defined as a > 30% decrease in the sum of the perpendicular diameters of all measurable lesions that was present for at least 4 weeks. Progressive disease (PD) was defined as either a > 20% increase in the sum of the products of measurable lesions over the smallest sum observed or the appearance of new lesions. Stable disease (SD) did not qualify as a CR, PR, or PD. Safety and adverse events were assessed according to the National Cancer Institute CTCAE (ver. 3.0).

### Statistical analysis

According to the Ogata et al.’s report [21], the setting dose of an ED during chemotherapy for colon cancer was 1 pack (80 g)/day, but the average amount of the ED that was able to be taken was 51.7%. This study is a triplet regimen for esophageal cancer, so the setting dose of an ED is 2 packs (160 g)/day. The sample size was calculated at 75% expected completion rate, 45% threshold completion rate with 80% detection power. The number of cases required was 20. We calculated the percentage of ED compliance for all registered patients. The differences between the 2 groups in ED compliance were analyzed using the Wilcoxon rank sum test {median [25th percentile (Q1), 75th percentile (Q3)]}. The factors affecting expression of OM ≥ Grade 2 were analyzed using logistic regression analysis. The digestive system adverse events were compared to those in the historical data of other report using Fisher’s exact test. *p* values of < 0.05 were considered to indicate statistical significance. All of the statistical analyses were performed using the SAS software program (ver. 9.4; SAS Institute Inc., Cary, NC, USA).

### Ethical considerations

This trial was conducted in accordance with the World Medical Association Declaration of Helsinki and was registered with the University Hospital Medical Information Network Clinical Trials Registry (Registration number: UMIN000010860). The study protocol was approved by the independent ethics committees of each of the four participating institutions, and written informed consent was obtained from all of the patients.

## Results

### Patients

Twenty patients were enrolled, 19 patients (one case was missing photographic data) were targeted as subjects for the analysis of the secondary endpoints (Table 1). The median age was 68 years (range 37–75 years). The performance status was 0 in 8 patients and 1 in 11 patients. The tissue types included squamous cell carcinoma (*n* = 18) and adenocarcinoma (*n* = 2).

**Table 1:** Patient characteristics in the feasibility study (*n* = 20)

	No. of patients	(%)
Age (years)		
Median (range)	68 (37-75)	
Sex		
Males/females	20/0	100/0
ECOG performance status		
0/1/missing data	8/11/1	40/55/5
Histological type		
Squamous cell carcinoma/adenocarcinoma	18/2	90/10
Site of primary tumor		
Ce/Ut/Mt/Lt/Ae	2/1/10/5/2	10/5/50/25/10
Differentiation		
Well/moderate/poor/unknown	6/10/2/4	30/50/10/20
Clinical T stage		
cT1b/T2/T3	2/4/14	10/20/70
Clinical N stage		
cN0/N1/N2/N3	1/8/8/3	5/40/40/15
Clinical stage		
II/III	4/16	20/80

*ECOG* Eastern Cooperative Oncology Group, *Ut* upper thoracic esophagus, *Mt* middle thoracic esophagus, *Lt* lower thoracic esophagus, *Ae* abdominal esophagus

### Compliance with the orally administered ED

Fourteen of the 20 patients [70%: 95% confidence interval (CI) 48.1–85.5%]= completed the orally administered ED (160 g/day; the completion group); 6 patients could not (non-completion group). Of these 6 patients, 3 (15%) could not take the ED orally for 9, 14, and 21 days, respectively, while 1 patient (5%) took the ED orally at an average dose of 80 g/day for 35 days. The remaining 2 patients (10%) could not take the 80 g/day dose for 11 and 12 days, respectively.

### The incidence of OM

Based on the results of the CRS, OM was observed in all 19 patients (100%), and grade ≥ 2 OM was found in 6 of the 19 patients (31.6%: 95% CI 12.6–56.6%). The grades of OM in the 19 patients were as follows: grade 1 (*n* = 13; 68.4%); grade 2 (*n* = 5; 26.3%), and grade 3 (*n* = 1; 5.3%).

The incidence of OM was as follows: grade 0 (*n* = 0; 0%), grade 1 (*n* = 11; 84.6%), grade 2 (*n* = 2; 15.4%), and grade 3 (*n* = 0; 0%) in the completion group (*n* = 13); and grade 0 (*n* = 0; 0%), grade 1 (*n* = 2; 33.3%), grade 2 (*n* = 3; 50%), and grade 3 (*n* = 1; 16.7%) in the non-completion group (*n* = 6).

The incidence of grade ≥ 2 OM in the ED completion group (15.4%; 2 of 13 patients) was significantly lower than that in the non-completion group (66.7%; 4 of 6 patients) (*p *= 0.046).

### The relationship between compliance with the ED and other parameters

No significant difference was observed between the two groups in the rate of body weight change during chemotherapy. Although the change in DAO activity during chemotherapy did not differ between the groups to a statistically significant extent, the change in DAO activity tended to be greater in the completion group [17.45 (− 1.55, 60.95)] than that in the non-completion group [− 23.00 (− 64.80, 15.60)], especially on Day 15 in cycle 1 (*p *= 0.1939) (Table 2). During the second cycle of chemotherapy, the prealbumin level in the completion group was significantly higher than that in the non-completion group (Day 1, *p *= 0.0037; Day 8, *p *= 0.0451). There was no significant difference between the groups with regard to the change in the lymphocyte count during chemotherapy (Table 2).

**Table 2:** Compliance with oral administration of ED and rates of change of each parameter Wilcoxon rank-sum test

	Cycle 1, Day 1	Cycle 1, Day 8	Cycle 1, Day 15	Cycle 2, Day 1	Cycle 2, Day 8	Cycle 2, Day 15
Body weight ΔMedian (Q1, Q3) (%)						
Completion group (*n *= 14)		− 0.05 (− 1.40, 0.90)	− 1.00 (− 1.90, 1.10)	0.70 (− 0.30, 2.15)	1.40 (− 0.45, 2.25)	0.75 (0.00, 2.70)
Non-completion group (*n *= 6)		− 1.45 (− 2.30, 0.00)	0.70 (− 3.40, 1.80)	0.85 (− 2.45, 3.05)	0.30 (0.00, 0.70)	− 1.45 (− 4.35, 0.50)
		*p* = 0.3219	*p* = 0.6294	*p* = 1.0000	*p* = 0.7517	*p* = 0.1290
DAO ΔMedian (Q1, Q3) (%)						
Completion group (*n *= 14)		− 10.30 (− 40.30, 16.90)	17.45 (− 1.55, 60.95)	27.60 (− 13.45, 50.35)	− 14.85 (− 25.25, 19.90)	9.35 (− 10.05, 38.50)
Non-completion group (*n *= 6)		− 17.10 (− 44.10, 32.60)	− 23.00 (− 64.80, 15.60)	9.40 (− 63.50, 17.50)	− 2.50 (− 98.40, 43.50)	20.20 (7.50, 32.90)
		*p* = 1.0000	*p* = 0.1939	*p* = 0.1939	*p* = 0.8852	*p* = 1.0000
Prealbumin ΔMedian (Q1, Q3) (%)						
Completion group (*n *= 14)		30.55 (5.90, 50.00)	0.00 (− 17.10, 13.00)	9.15 (− 4.55, 24.50)	55.50 (20.80, 68.40)	15.60 (5.80, 46.55)
Non-completion group (*n *= 6)		15.35 (− 31.30, 36.80)	− 19.40 (− 37.50, − 16.70)	− 24.10 (− 33.30, − 9.70)	− 3.30 (− 5.30, 6.50)	1.15 (− 32.85, 47.25)
		*p* = 0.2831	*p* = 0.0745	*p* = 0.0037^※^	*p* = 0.0451^※^	*p* = 0.6276
Lymphocytes ΔMedian (Q1, Q3) (%)						
Completion group (*n *= 14)		− 2.25 (− 11.60, 0.40)	− 8.70 (− 255.70, − 3.20)	− 8.55 (− 12.60, 1.20)	− 5.85 (− 20.10, 8.45)	− 8.25 (− 15.70, 14.30)
Non-completion group (*n *= 6)		− 6.60 (− 22.30, 0.60)	− 17.80 (− 24.30, − 8.80)	− 9.25 (− 30.40, 6.20)	− 7.00 (− 20.80, 1.00).	− 11.65 (− 29.90, 0.95)
		*p* = 0.6207	*p* = 0.4829	*p* = 0.7079	*p* = 0.7518	*p* = 0.4669

^※^*p* < 0.05

### Adverse events other than OM

The grade 3 adverse events were as follows: fatigue (*n* = 3; 15%), fever (*n* = 3; 15%), anorexia (*n* = 3, 15%); diarrhea (*n* = 2; 10%), and nausea (*n* = 1; 5%) (Table 3). One patient in the non-completion group died due to sudden cardiac arrest after 2 cycles of chemotherapy; the patient’s death was probably related to DCF toxicity. The incidence of grades 1–3 anorexia was as follows: grade 0 (*n* = 4; 28.6%), grade 1 (*n* = 5; 35.7%), grade 2 (*n* = 5; 35.7%), and grade 3 (*n* = 0; 0%) in the completion group (*n* = 14); and grade 0 (*n* = 0; 0%), grade 1 (*n* = 2; 33.3%), grade 2 (*n* = 1; 16.7%); and grade 3 (*n* = 3; 50%) in the non-completion group (*n* = 6). grade ≥ 2 anorexia occurred in 5 of the 14 patients (35.7%) in the completion group and in 4 of the 6 patients (66.7%) in the non-completion group (*p *= 0.3359).

**Table 3:** All adverse events excluding the oral mucositis (*n *= 20)

	Grade				All grades *n* (%)	≥ Grade 2 *n* (%)	≥ Grade 3 *n* (%)
	1	2	3	4			
Hearing disturbance	0	0	0	0	0 (0)	0 (0)	0 (0)
Fatigue	4	3	3	0	10 (50)	6 (30)	3 (15)
Fever	2	1	3	0	6 (30)	4 (20)	3 (15)
Alopecia	13	3	–	–	16 (80)	3 (15)	–
Pigmentation	0	1	0	0	1 (5)	1 (5)	0 (0)
Skin rash	0	0	0	0	0 (0)	0 (0)	0 (0)
Cutaneous symptoms of the hands and feet	0	0	0	0	0 (0)	0 (0)	0 (0)
Anorexia	7	6	3	0	16 (80)	9 (45)	3 (15)
Constipation	0	0	0	0	0 (0)	0 (0)	0 (0)
Diarrhea	7	1	2	0	10 (50)	3 (15)	2 (10)
Nausea	3	2	1	0	6 (30)	3 (15)	1 (5)
Infection (accompanied by neutropenia)	0	0	6	0	6 (30)	6 (30)	6 (30)
Edema	1	1	0	0	2 (10)	1 (5)	0 (0)
Neuropathy (motor)	0	0	0	0	0 (0)	0 (0)	0 (0)
Neuropathy (sensory)	1	0	0	0	1 (5)	0 (0)	0 (0)
Watery eyes	0	0	0	0	0 (0)	0 (0)	0 (0)
Leucopenia	–	9	6	3	–	18 (90)	9 (45)
Neutropenia	–	3	10	5	–	18 (90)	15 (75)
Anemia	–	2	0	0	–	2 (10)	0 (0)
Thrombocytopenia	–	0	0	0	–	0 (0)	0 (0)

### The objective response rate to chemotherapy

The objective response rate to chemotherapy was 66.7% [CR, *n* = 2 (11.1%); PR, *n* = 10 (55.6%); SD, *n* = 6 (33.3%); and PD, *n* = 0 (0%)]. The responses in the completion group were CR [*n* = 1 (8.3%)], PR [*n* = 7 (58.3%)], SD [*n* = 4 (33.3%)], and PD [*n* = 0 (0%)]. The responses in the non-completion group were CR [*n* = 1 (16.7%)], PR [*n* = 3 (50%)], SD [*n* = 2 (33.3%)], and PD [*n* = 0 (0%)]. There was no significant difference between the patients who could and could not complete the ED (*p *= 1.000). Surgical resection was performed in 18 patients. One patient selected observation rather than surgery because a CR was attained, and 1 patient died after chemotherapy. No postoperative complications were observed, and the administration of the ED did not interfere with any of the planned operations.

## Discussion

In the present study, 14 of the 20 patients (70%) completed taking an orally administered ED at a dose of 2 packs (160 g)/day during chemotherapy.

Besides taste and satiety, there may be several reasons why the orally administered ED was or was not completed. First, there is likely to be a difference in the completion rate due to anorexia. Although there was no significant difference between the loss or withdrawal of the ED and anorexia, a relationship between these factors was suggested in the present study. Second, the patients in whom the antitumor effect was poor, and in whom stenosis worsened, could not take the ED. However, there was no significant difference in the objective response rate to chemotherapy between the patients who could and could not complete the ED. Third, there is likely to be a relationship between poor compliance and the development of oral pain from OM. We did not refrain from administering analgesics. In fact, 2 patients (15.4%) with grade ≥ 2 OM completed the oral administration of the ED, and 2 patients (33.3%) without grade ≥ 2 OM could not complete the oral administration of the ED. Still, we are of the opinion that it is important to consider administering an ED with DCF chemotherapy. Although the number of cases was small, the incidence of OM in the ED completion group was significantly suppressed in comparison to the non-completion group.

Nishimura et al. reported that the incidence of OM (grade ≥ 1) was the highest during chemotherapy for breast cancer (76.5%), followed by head and neck cancer (67.7%), colorectal cancer (63%), and esophageal cancer (57.8%). When classified by chemotherapy regimen, the incidence of OM (grade ≥ 1) was the highest among those receiving DCF (85.7%), followed by those receiving 5-FU/leucovorin/irinotecan (80%) and 5-FU/cyclophosphamide/adriamycin (78.8%). Moreover, the incidence of grade ≥ 2 OM among patients receiving DCF was approximately 40% [22].

The exact objective incidence of OM may not be known because its incidence is described according to complaints of the patient or assessment by general physicians or medical staff members who are not specialists in the oral environment; thus, its incidence may often be underestimated. A thorough examination of the intraoral condition with instruments specific to the oral cavity can only be conducted by oral and maxillofacial surgeons, dentists, and their teams. We therefore constructed the CRS to assess the oral environment.

The grade ≥ 2 OM rate in previous DCF report was 28% [10] and all the grade ≥ 2 OM rate in this study was 31.6%. In our previous study including FP and triplet regimen, OM of grade ≥ 2 was occurred in 10% with Gln plus an ED group [17]. The incidence of grade ≥ 2 OM in the 2 packs (160 g/day) of ED completion group was in 15.4% that was significantly lower than non-completion group. Although the OM suppression effect in this study seems to be low, it is doubtful whether the oral cavity was completely evaluated in those previous studies.

In this study, based on the results of the CRS, OM of Grade ≥ 1 was actually observed in all patients (100%). Thus, the judgment of OM by general clinicians might be lower than that by dental specialists, which suggests that in the clinical setting the actual incidence of OM among patients undergoing chemotherapy for cancer may be greater than clinicians realize.

For this reason, we focused on the following characteristics of EDs. An ED is a specialized formula containing a blend of proteins as amino acids. Because of its nature, little digestion is necessary, and it shows high absorption efficiency. Thus, EDs are frequently used for patients with inflammatory bowel disease, in particular patients with Crohn’s disease (CD).

The effects of EDs in CD have been widely reported [18, 23]; in particular, the induction of remission [24] and sustained remission [25] from CD has been reported. An ED has been shown to have a clear suppressive effect on clinical activity and on inflammatory cytokines such as interleukin (IL)-1β, IL-6, and tumor necrosis factor-α (TNF-α) [23]. Moreover, histidine inhibited the production of TNF-α and IL-6 by mouse macrophages [18]. Current studies have shown that amino acids themselves can protect the mucosa and have anti-inflammatory effects [18, 26]. The administration of an ED during cancer chemotherapy has been reported to have the potential prevent OM [17, 21]. Chemotherapy damages DNA through the production of reactive oxygen species, the induction of apoptosis through the upregulation of the expression of intracellular molecules, and the production of several cytokines, such as IL-1β, IL-6, and TNF-α [27, 28].

Because OM is reported to be caused by chemotherapy-induced mucosal damage [17], we measured the mucosal integrity on the basis of DAO activity. As a result, we found that the integrity of the intestinal mucosa tended to be maintained in the ED completion group. The previous report also showed that chemotherapy reduced the integrity of the intestinal mucosa and that a combination of an ED and Gln maintained the integrity to a significantly greater extent than Gln alone during chemotherapy, indicating a possible connection with the environment of the oral cavity [17]. Previous reports have shown that amino acids might be more absorbable—even during chemotherapy—from the viewpoints of efficacy in maintaining the mucosal integrity and their easy digestibility. In addition it has been hypothesized that an ED might also offer a mucosal protective effect in chemotherapy-induced mucositis via mechanisms that are similar to those that provide a suppressive effect against inflammatory cytokines in CD.

The high completion rate of the orally administered 2 packs of ED (160 g/day) suggested the possibility of decreased OM. The factors that may affect expression of grade ≥ 2 OM were compared, but no significant difference was observed between the two population except compliance of ED (Table 4).

**Table 4:** Logistic regression analysis of factors affecting expression of oral mucositis ≥ Grade 2

Factors	Oral mucositis ≥ Grade 2		Odds ratio (95% CI)	*p* value
	−	+		
Age				
< 70	6	3		
≥ 70	5	3	1.20 (0.16–8.80)	0.8577
Performance status				
0	5	2		
1	7	4	1.43 (0.18–11.09)	0.7330
Body mass index				
< 22	6	3		
≥ 22	7	3	0.86 (0.12–5.94)	0.8760
Histopathology				
Squamous cell carcinoma	12	5		
Adenocarcinoma	1	1	2.40 (0.12–46.39)	0.5624
Location				
Upper Middle esophagus	8	4		
Lower esophagus	5	2	0.80 (0.10–6.10)	0.8296
Macroscopic type				
Bulging type	2	1		
Ulceration type	11	5	0.91 (0.07–12.52)	0.9432
Wall depth degree				
T1, T2	2	3		
T3	11	3	0.18 (0.02–1.64)	0.1285
Lymph node metastasis				
N0, N1	6	2		
N2 ,N3	7	4	1.71 (0.23–12.89)	0.6006
Cancer stage				
II	1	2		
III	12	4	0.17 (0.01–2.37)	0.1857
Underlying disease				
Negative	7	1		
Positive	6	5	5.83 (0.52–64.79)	0.1512
Past illness				
Negative	5	4		
Positive	6	2	0.42 (0.05–3.31)	0.4074
Albumin (g/dl)				
≤ 3.7	6	1		
> 3.7	7	5	4.29 (0.39–47.62)	0.2362
Prealbumin (mg/dl)				
≤ 20	8	1		
> 20	5	5	8.00 (0.71–90.00)	0.0922
Retinol binding protein (mg/dl)				
≤ 3	6	1		
> 3	7	5	4.29 (0.39–47.62)	0.2362
Ferritin (ng/ml)				
≤ 100	3	3		
> 100	10	3	0.30 (0.04–2.34)	0.2510
Transferrin (mg/dl)				
≤ 200	4	1		
> 200	9	5	2.22 (0.19–25.72)	0.5228
CRP (mg/dl)				
≤ 0.3	7	3		
≫ 0.3	6	3	1.17 (0.17–8.09)	0.8760
Plasma diamine oxidase activity (U/ml)				
≤ 5	8	1		
> 5	3	4	10.67 (0.82–138.22)	0.0701
IgA (mg/dl)				
≤ 200	6	2		
> 200	6	4	2.00 (0.26–15.38)	0.5056
Compliance of 160 g/day of elemental diet				
Non-completion	2	4		
Completion	11	2	0.09 (0.01–0.88)	0.0384^※^

^※^*p* < 0.05

In the present study, the combination of the ED with esophageal cancer chemotherapy did not increase the rate of adverse digestive events in comparison to the historical data of another report on DCF therapy [8] (Table 5).

**Table 5:** Comparison of digestive adverse events with historical data Fisher’s exact test

	EPOC study (*n *= 20)		Historical data^a^ (*n *= 42)		*p* value	
	All grade	Grade 3 ≤	All grade	Grade 3 ≤	All grade	Grade 3 ≤
Anorexia	16 (80%)	3 (15%)	39 (92.9%)	3 (7.1%)	0.1986	0.3773
Diarrhea	10 (50%)	2 (10%)	16 (38.1%)	0 (0%)	0.4186	0.1005
Nausea	6 (30%)	1 (5%)	28 (66.7%)	0 (0%)	0.013	0.3226

^a^Hara et al. [8]

The intention behind initiating the oral intake of the ED from 1 week before chemotherapy was to prevent the occurrence of pain from OM. Without the pain of OM, patients can eat regular meals and continue taking the ED through the chemotherapy cycle. In addition, when enteral nutrients are administered orally, poor compliance due to taste becomes a serious problem. Flavoring agents or a jelly mix may be good choices to make it easier for the patient to accept the taste of the ED. Measures against satiety are also important. By avoiding both increases in the caloric intake up to 1 h before a meal and uncontrolled increases in blood sugar throughout the day [29], the patients who received the ED over a period of 3 h at the same start time for breakfast and dinner tended to be able to receive the ED at a dose of 160 g/day throughout the course of chemotherapy in the present study. Another report also showed the possibility of orally administering an ED at a dose of 160 g/day [30].

The present study is associated with several limitations. First, it would be preferable to use more than one judge to assess OM in the CRS. Second, compliance to the ED should be investigated in two groups in a larger-scale study. In the present study, we wanted to determine how much of the ED could be administered orally to patients undergoing DCF chemotherapy and to investigate the differences in physiological activity according to compliance to the ED. Thus, we set the dose of the ED to 160 g/day and assessed patient compliance, adverse events, and other parameters for use in a future phase III study.

## Conclusion

Our multi-institutional study revealed that 14 of 20 (70%) patients with esophageal cancer completed the oral administration of an ED at a dose of 2 packs (160 g/day) during DCF chemotherapy. The CRS was useful for determining the precise incidence of OM. An ED might be a one of the test treatment to reduce the incidence of OM and should be evaluated in further randomized study. We have, therefore, begun a prospective multi-institutional phase III trial using the CRS.

## Electronic supplementary material

Below is the link to the electronic supplementary material.

Supplementary material 1 (TIFF 605 kb)
