# Supplementary figures and images for: The effect of an elemental diet on oral mucositis of esophageal cancer patients treated with DCF chemotherapy: a multi-center prospective feasibility study (EPOC study)
Source: Esophagus. 2018 May 31;15(4):239–48. doi: 10.1007/s10388-018-0620-1 (PMC6153973; doi:10.1007/s10388-018-0620-1)

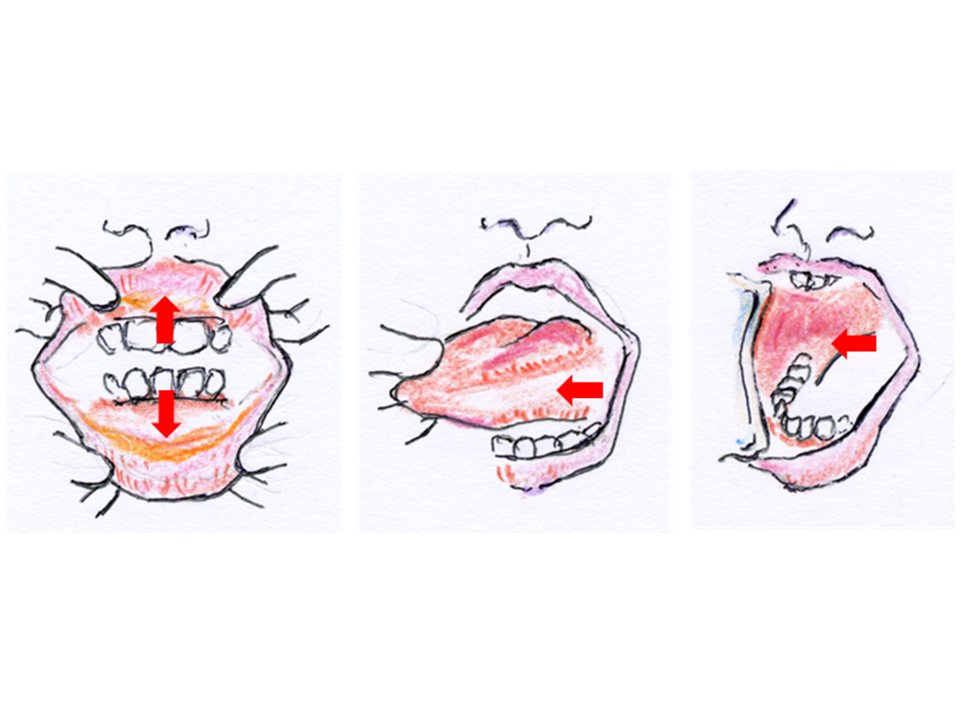

Supplement: Supplementary file 1 — Supplementary material 1 (TIFF 605 kb) [file 10388_2018_620_MOESM1_ESM.tiff]
